# Mapping the Incidence of Infestation by *Neoechinorhynchus buttnerae* (Acanthocephala) Parasitizing *Colossoma macropomum* Raised in Fish Farms and the Relationship with Zooplankton Ostracods and Copepods

**DOI:** 10.3390/vetsci12010006

**Published:** 2024-12-29

**Authors:** Vinicius Perez Pedroti, Jerônimo Vieira Dantas Filho, Átila Bezerra de Mira, Maria Mirtes de Lima Pinheiro, Bruna Lucieny Temponi Santos, Raniere Garcez Costa Sousa, Jucilene Braitenbach Cavali, Ed Johnny da Rosa Prado, Sandro de Vargas Schons

**Affiliations:** 1 Grupo de Pesquisa em Patologia Animal no Bioma Amazônico, Centro de Diagnóstico Animal, Programa de Pós-Graduação em Ciências Ambientais, Universidade Federal de Rondônia, Rolim de Moura 76940000, Brazil; pedroti.peixe@gmail.com (V.P.P.); sandroschons@unir.br (S.d.V.S.); 2 Curso de Medicina Veterinária, Grupo de Estudo e Pesquisa em Biomonitoramento Ambiental, Centro Universitário São Lucas Ji-Paraná Afya, Ji-Paraná 76907524, Brazil; 3 Curso de Geografia, Universidade Federal de Rondônia, Porto Velho 76801058, Brazil; ranieregarcez@gmail.com; 4 Curso de Engenharia de Pesca, Universidade Federal de Rondônia, Presidente Médici 76916000, Brazil; 5 Programa de Pós-Graduação em Sanidade e Produção Animal Sustentável na Amazônia Ocidental, Universidade Federal do Acre, Rio Branco 69920900, Brazil

**Keywords:** Brazilian Amazon, environmental monitoring, fish farming, fish parasitology, georeferencing

## Abstract

This study investigated the abundance of zooplankton copepods and ostracods taxonomic groups in fish farms in Rondônia’s Vale do Jamari and Centro-Leste microregions during the rainy and dry seasons. It also analyzed the correlation between zooplanktons abundance and the presence of acanthocephalan parasites. Conducted in 41 fish farms between November 2021 and September 2023, the study included water, zooplankton, and fish samples from 196 *Colossoma macropomum*. The results showed that 95% of the farms had fish infected with *Neoechinorhynchus buttnerae*, with varying parasitism levels. Georeferencing revealed higher parasite densities in the municipalities of Ariquemes, Monte Negro, Machadinho do Oeste, and Buritis (Vale do Jamari), as well as Urupá, Ji-Paraná, Ouro Preto do Oeste, and Teixeirópolis (Centro-Leste), with clusters of heat islands in the latter group. Water quality parameters were suitable for raising *C. macropomum*. The presence of ostracods and copepods could serve as indicators of parasitic infestations, highlighting the importance of monitoring zooplankton and parasite communities. This approach is valuable for detecting changes in artificial ecosystems, such as fish farms, which could lead to significant long-term effects.

## Simple Summary

This study examined the abundance of zooplankton groups (copepods and ostracods) and their correlation with acanthocephalan parasites in fish farms across Rondônia’s Vale do Jamari and Centro-Leste regions during rainy and dry seasons. Conducted in 41 fish farms from November 2021 to September 2023, it included water, zooplankton, and fish samples from 196 *Colossoma macropomum*. Results revealed that 95% of farms had fish infected with *Neoechinorhynchus buttnerae*, with higher parasite densities in municipalities like Ariquemes and Ji-Paraná. Water quality was suitable for fish farming. Zooplankton presence, particularly copepods and ostracods, correlated with parasitic infestations, suggesting their potential as ecological indicators. Monitoring these communities is critical for detecting changes in artificial ecosystems, such as fish farms, to mitigate long-term effects.

## 1. Introduction

According to the FAO report [1], in the year 2020, global seafood production reached 214 million tons, with an estimated value of USD 406 billion. Of this production, marine fishing accounted for 78.8 million tons, while freshwater fishing contributed 11.5 million tons. On the other hand, aquaculture animal production reached a new milestone, producing 87.5 million tons of seafood worldwide. This represented a 4.4% decrease in captures and a 5.7% increase in aquaculture. Brazil ranked 13th globally, with 630 thousand tons, placing 8th in terms of largest inland fish production with 552 thousand tons. The state of Rondônia stands out as the main producer of native fish varieties in Brazil, with *Colossoma macropomum* leading this production [2,3].

Intensification in cultivation may favor the emergence of diseases in this species, such as acanthocephaliasis caused by *Neoechinorhynchus buttnerae* (Golvan, 1956) (Acanthocephala: Neoechinorhynchidae), an obligate endoparasite observed in the intestines of fish. It can cause damage and/or obstruction of the intestinal lumen, leading to productivity loss and death. Clinical signs include cachexia, growth retardation, decreased mucus production, drying of the body surface, dull skin, and disproportion between the head and body, with considerable muscle mass loss in the dorsal region [4].

In aquatic environments, fish are subject to various environmental fluctuations, causing stress and making them vulnerable to pathogens. Studies indicate that the most researched taxa include the groups Protozoa, Myxozoa, Monogenea, and Crustacea, as well as Digenea, Cestode, Nematoda, and Acanthocephala. The latter are endoparasites, frequently found in studies related to the environment, especially those with heteroxenous life cycles, which show population changes related to the death of intermediate hosts [5].

The lack of sanitary maintenance in cultivation ponds, particularly concerning water quality, can promote an environment favorable to the life cycle of unwanted organisms [6]. Parasites like acanthocephalans may benefit from this condition, ensuring their perpetuation through eggs deposited in organic matter or other aquatic microorganisms (ostracods and copepods), which harbor the juvenile form of the parasite (acanthella). According to Malta et al. [7], the phylum Acanthocephala consists of helminths that are exclusively intestinal parasites of vertebrates, with nine species occurring in Amazonian fish, two of which are capable of parasitizing *C. macropomum* as follows: *Echinorhynchus jucundus* (Travasses, 1923) from the Echinorhynchidae family, and *Neoechinorhynchus buttnerae* Golvan, 1956 from the Neoechinorhynchidae family (Fischer, 1998). The same authors also state that Ostracoda, Copepoda, and Megaloptera larvae are the intermediate hosts of acanthocephalans from the Neoechinorhynchidae family.

Based on the above, this study aims to determine the incidence and abundance of acanthocephalan infection in *C. macropomum* in two microregions of Rondônia state (Brazil), as well as the correlations between infection intensity, zooplankton populations, water quality, control and prevention practices, and the use of chemical antiparasitics to control parasitosis.

## 2. Material and Methods

### 2.1. Ethical Considerations and Study Area

The study was conducted after authorization from the Research Ethics Committee (CEP/CONEP) of the Universidade Federal de Rondônia, Brazil. Plataform Brazil assigned CAAE authorization protocol No. 60744322.5.0000.5300 and receipt No. 077412/2022. The title of the approved research project is “Health characterization of fish farming in Rondônia state”.

**Figure 1:**
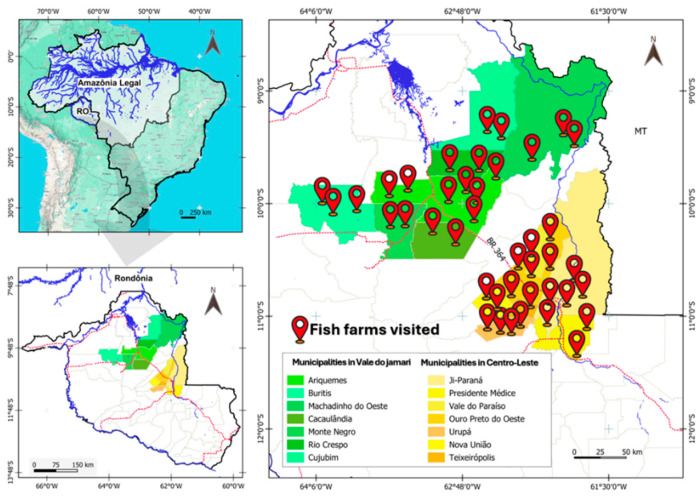
Geographic location of the fish farms visited in Rondônia; yellow municipalities located in the Central-East microregion, and green municipalities located in the Vale do Jamari microregion.

The study of parasitic fauna was carried out in 41 fish farms while the zooplanktonic community of ostracods and copepods was analyzed in the water of excavated ponds of 30 fish farms, in the microregions of Vale do Jamari and Centro-Leste, in the state of Rondônia, Brazil. These fish farms visited are in the municipalities of Ariquemes, Buritis, Cacaulandia, Cujubim, Machadinho D’ Oeste, Monte Negro, and Rio Crespo (in the Vale do Jamari); and, in municipalities of Ji-Paraná, Urupá, Ouro Preto do Oeste, Teixeirópolis, Nova União, Vale do Paraíso, Mirante da Serra, and Presidente Médici (in the Centro-Leste) (Figure 1).

The research was conducted during the Amazon hydrological seasons, rainy (November to March) and dry (April to October), from November 2021 to September 2023. Fish farms were randomly selected among the 550 fish farms registered in the Healthy Fish Program, conducted by technicians from the Technical Assistance and Rural Extension Authority of the State of Rondônia (Technical Assistance and Rural Extension Management System-SIGATER). All visited farms raised *Colossoma macropomum* in a semi-intensive system in fishponds (excavated tanks).

The climate of the state of Rondônia is classified by the Köppen system as predominant type Am—Rainy Tropical Climate (megathermal) [8], with a well-defined dry period characterized by a moderate water deficit and rainfall levels below 50 mm /month. The annual average rainfall varies between 1400 and 2600 mm/year, while the monthly average air temperature varies between 24 and 26 °C during the rainy season, reaching 36 °C during the dry season [9].

### 2.2. Quali–Quantification of Zooplankton and Physicochemical Analyzes

Water and zooplankton samples were collected from fishponds. The collections were carried out in triplicate using a completely randomized factorial design 30 × 3 × 3 (30 fish farms, 3 fishponds, and 3 repetitions). Sampling locations were selected at three points in each fishpond as follows: upstream, at the drainage pipe (outflow), and at the water column between the other two points.

For selecting sampling points, the water supply for fishponds in production where fish were being cultivated was considered. Water and zooplankton samples were collected during the two Amazonian hydrological seasons. Following the suggestion by Costa et al. [10], ponds connected in a series, where water from the supply reservoir feeds the first pond and then flows into the next, alternating fishponds sampling was performed. The geographic coordinates of the collection points were recorded using the Global Positioning System (GPS).

To obtain qualitative samples, horizontal and vertical tows were performed on the water surface of the ponds, the supply reservoir, and the effluent. Each quantitative sample was obtained using a plankton net (with a 50 μm mesh), and a graduated bucket was used to collect the water. Since the focus of this study was zooplankton, the samples were immediately stored and sedimented in polyethylene terephthalate bottles and kept at 7 °C in cooler-type thermal boxes until being sent to the laboratory [11,12]. The biological material was sedimented, filtered, and preserved in a 6:3:1 solution, equivalent to 60% distilled water, 30% ethyl alcohol, and 10% formaldehyde. Then, five drops of 10% copper sulfate were added to each sample to preserve the coloration of the organisms to be observed [11,12].

For qualitative–quantitative analyses, zooplankton abundance was expressed in individuals per 100 mL (Ind. 100 mL^−1^). A total of 540 water samples were examined [(30 × 3 × 3) × 2 seasons)] using a micropipette (calibrated at 1 mL), with 2.0 mL subsamples taken for counting individuals in a Neubauer chamber and identification using a Bioval binocular optical stereoscopic microscope (Sigma, Livonia, MI, USA), with a magnification capacity of up to 10^3^×. The microscope was equipped with a professional digital camera (Canon EOS Rebel T8i EF-S 18–55 mm). Photomicrographs of the images obtained were then amplified using ViPlus software, which validates morphological and behavioral identification. The database with the taxonomic keys used was from Hernández et al. [13] and Mindat (https://www.mindat.org/, accessed on January to May 2024).

### 2.3. Parasitic Fauna: Incidence and Infestation

Epidemiological data were collected through a semi-structured questionnaire from rural producers or those responsible, with questions related to fish production, ways to control endoparasitosis, and sanitary measures applied in fish farming. These collected epidemiological data were entered and analyzed in the Epi info^TM^ software database, version 3.5.3—2011 (OS: MS-Windows, C Sharp programming language) and descriptive statistical analysis was performed.

Table 1 shows the survey of parasitic fauna in 41 fish farms, for 11 of which it was not possible to collect water, so 30 fish farms were considered for the zooplankton inventory. Furthermore, the number of fish collected per property as well as the average weight (g) and total length by hydrological seasons are shown. Using a fishing rod with line and hook, 196 *C. macropomum* were captured, 96 during the rainy season and 100 during the dry season (Table 1).

A total of 3 fish were caught per fish farm (out of the 41 properties) in each season; therefore, a total of 123 fish were caught in the dry season and 123 fish in the rainy season.

The fish were examined, and the laboratory analyses were carried out at the Animal Diagnostic Center (CDA) at the Universidade Federal de Rondônia (UNIR), Rolim de Moura Campus, Brazil. Following the methodologies of Lucena et al. [6], fish previously anesthetized in eugenol solution (40 mL L^−1^) had their total length and total weight measured. Anesthetized animals were euthanized by spinal transection. An abdominal incision was then made to remove the organs from a bloc. The liver, spleen, stomach, and intestines were separated, desiccated, and placed in Petri dishes. The livers and spleens were weighed on a digital scale with a minimum sensitivity of 0.01 g and a maximum sensitivity of 300 g, with values recorded on individual cards per animal, and the allometric Kn condition factor (ka) was subsequently calculated (Equation (1)). The hepatosomatic ratio, HSI (%), is represented by Equation (2), and the splenosomatic ratio, SSI (%), is represented by Equation (3).

k_a_ = W/L^b^ (1)

where W = total weight, L = Length and b = angular coefficient of the weight/length relationship).

HSI (%) = liver weight (g)/body weight (g) × 100(2)

SSI (%) = spleen weight (g)/weight body (g) × 100(3)

The intestines and stomach were placed in a Petri dish containing cold water to immobilize the parasites. Subsequently, the stomach was separated from the intestine and the intestines were divided into anterior and posterior intestines. The viscera were dissected longitudinally under a stereomicroscope to remove and count parasites in the lumen and/or attached to the organ wall. The Acanthocephala were identified based on morphology and the presence of a small proboscis in relation to the body, as described by Virgilio et al. [14] and Lourenço et al. [15], and they were classified as the species *Neoechinorhynchus buttnerae* of the Neoechinorhynchidae family, specific to *C. macropomum*.

Parasite indices such as prevalence (p), intensity (I), and mean infection intensity (IMI) and abundance (A) were calculated on the number of fish collected (196) and were subsequently divided into subgroups by weight. To this end, the weight classification of 100–500 g for juveniles, 501–1500 for adults, and above 1500 ready for commercialization was used.

### 2.4. Statistical Analyzes

After being organized, the data were subjected to descriptive statistical analysis. Frequency analyses (%), arithmetic mean (μ), standard deviation (σ), and total range were employed for the better visualization of the results. Then, the data were submitted to the Shapiro–Wilk test (α = 0.05) to verify whether the samples came from a population with a normal distribution (H_0_). A Student’s *t*-test (at a 5% significance level) was then applied to compare zooplankton abundances, considering spatial variation (microregions of Rondônia state) and seasonality.

After confirming the normality and homoscedasticity of the data, mean tests and Pearson’s correlations were applied. Tukey’s test (at a 5% significance level) was used to compare (i) the morphometric variations, organ weights, hepatosomatic index (HSI), splenosomatic index (SSI), and condition factor (Kn); (ii) the prevalence, mean parasitic intensity, and abundance of parasites in *C. macropomum* at different farming stages—juveniles (100 to 500 g), adults (501 to 1500 g), and ready for commercialization (above 1500 g); and (iii) the means of the physicochemical parameters of aquaculture water across different microregions and seasons.

Additionally, correlations were made between (i) parasitic load, fish morphometry, and zooplankton abundance; (ii) *C. macropomum* weights and lengths, condition factors, and number of parasites; (iii) sanitary management practices and the number of recorded parasites; and (iv) the efficacy of anthelmintics/anti-parasitics, parasitic intensity, and water quality.

An overlap analysis was carried out between zooplankton populations and Acanthocephalus parasites. Abundances of copepod and ostracod species (Ind. L^−1^) under the conditions of the parasitic intensities—non-intense (<300 acanthocephalans per *Colossoma macropomum*) and intense (>300 acanthocephalans per *C. macropomum*)—were considered for the two microregions of Rondônia state and the two hydrological stations.

The Kernel heatmaps georeferencing methodology integrates statistical analyses with spatial data visualization to interpret complex ecological and aquaculture datasets. Regarding the parasite density illustrated on the heatmaps as heat islands, it is shown at densities of 10, 100, and 500 ha^−1^.

Pearson’s correlations highlighted relationships between biological variables, parasite metrics, and environmental factors. These results were georeferenced to produce Kernel heatmaps, visualizing data density and trends spatially. Heatmaps revealed patterns of parasite distribution and environmental variability, linked to farming stages and water quality across microregions and seasons. This methodology supports targeted management strategies in aquaculture, identifying hotspots for intervention and guiding resource allocation. By combining robust statistical tools with geospatial analysis, this approach provides a comprehensive framework for optimizing fish health and aquaculture sustainability.

All results were considered statistically significant with *p* < 0.05. Statistical analyses were performed using RStudio Development Core Team, version 3.5.3.

## 3. Results

### 3.1. Parasitic Fauna, Fish Health, and Environmental Conditions

Of the 41 properties visited, almost all had fish infected by *Neoechinorhynchus buttnerae* (Acanthocephala), with different intensities of parasitism. Fish farms P8, P25, and P38 were negative for the presence of the parasites during the rainy season and properties P1, P21, P23, and P34 during the dry season (Table 2).

**Figure 2:**
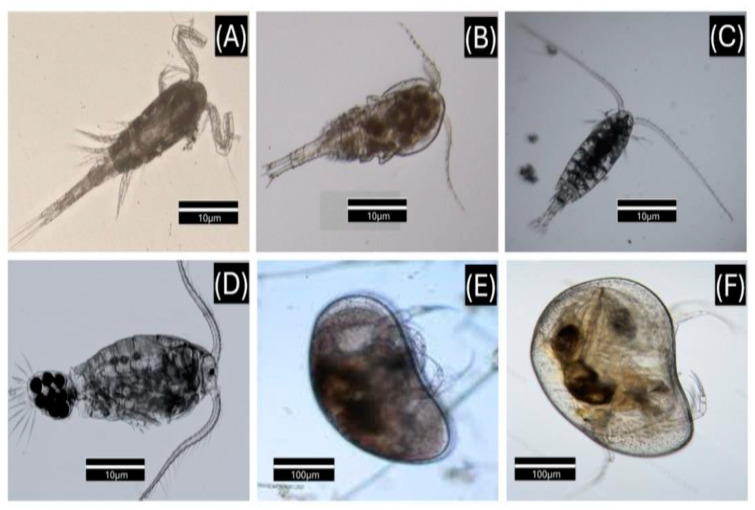
Photomicrographs of copepod species—Thermocyclops decipiens (**A**), *Acanthocyclops* sp. (**B**), *Argyrodiaptomus* sp. (**C**), and *Argyrodiaptomus furcatus* (**D**)—and ostracod species—*Heterocypris* sp. (**E**), and *Heterocypris punctata* (**F**).

Figure 2 illustrates copepod species—*Thermocyclops decipiens* (A), *Acanthocyclops* sp. (B), *Argyrodiaptomus* sp. (C), and *Argyrodiaptomus furcatus* (D)—and ostracod species—*Heterocypris* sp. (E) and *Heterocypris punctata* (F)—found in the freshwater of fish farms located in the interior of Rondônia state, Brazil.

An overlap analysis was carried out between zooplankton populations and Acanthocephalus parasites. Zooplankton copepods were more abundant in the water of fish farms in the Centro-Leste microregion (*p* < 0.05; 1046.27 Ind. L^−1^) with greater parasitic intensity (>300; 885.24 acanthocephalans per *C. macropomum*). While zooplankton ostracods were more abundant in the water of fish farms in the Vale do Jamari microregion (*p* < 0.05) with greater parasite intensity (>300; 764.31 acanthocephalans per *C. macropomum* (Table 3).

**Figure 3:**
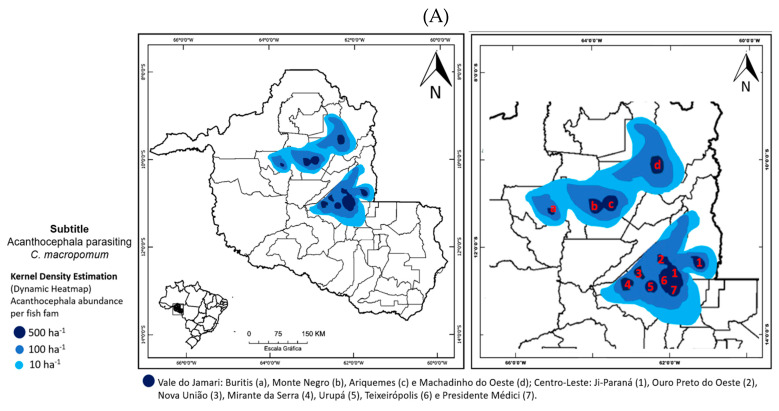

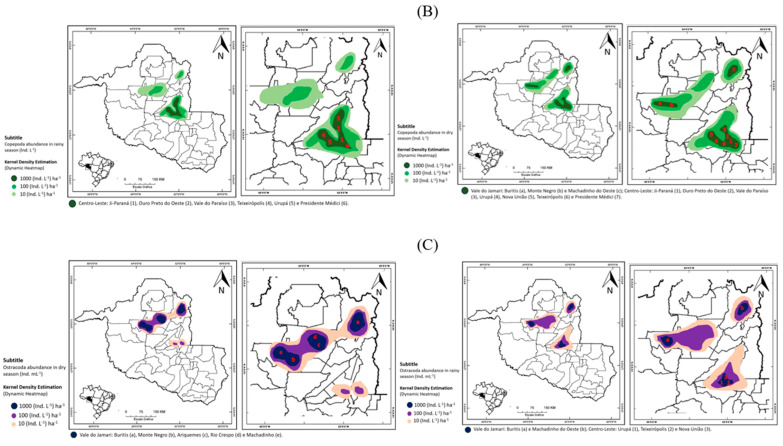
Maps of the Kernel Diversity Index by microregions and by season of records of *Neoechinorhynchus buttnerae* (Acanthocephala) parasitizing *Colossoma macropomum* (**A**), abundance of Copepoda (**B**), and Ostracoda (**C**) in the water of fish farms.

The georeferencing of acanthocephalan parasitic infestations in fishponds from Rondônia state, is showed through maps with Kernel heat islands. These maps showed that, in the Vale do Jamari microregion, the highest densities (in simulated graphical estimates) of acanthocephalan parasitic infestations are concentrated in the municipalities of Ariquemes (500 ha^−1^ in 380 km^2^), Monte Negro (500 ha^−1^ in 336 km^2^), Machadinho do Oeste (500 ha^−1^ in 331 km^2^), and Buritis (500 ha^−1^ in 99 km^2^) (Figure 3 A). In the Centro-Leste microregion, the highest densities (in simulated graphical estimates) are found in the municipalities of Urupá (500 ha^−1^ in 492 km^2^), Ji-Paraná (500 ha^−1^ in 410 km^2^), Ouro Preto do Oeste (500 ha^−1^ in 393 km^2^), Mirante da Serra (500 ha^−1^ in 387 km^2^), and Teixeirópolis (500 ha^−1^ in 198 km^2^) (Fugue 3B).

Notably, parts of the municipalities of Ji-Paraná, Ouro Preto do Oeste, Urupá, and Teixeirópolis displayed a cluster of heat islands with a density of 500 ha^−1^ across 889 km^2^. Zooplanktons, particularly copepods, were predominant in Centro-Leste (r = 0.83; *p* = 0.016), while ostracods were more common in the fishponds of the Vale do Jamari region (r = 0.89; *p* = 0.022) (Figure 3C). Acanthocephalans were more abundant in the Vale do Jamari (r = 0.98) compared to the Centro-Leste region (r = 0.89). 

**Figure 4:**
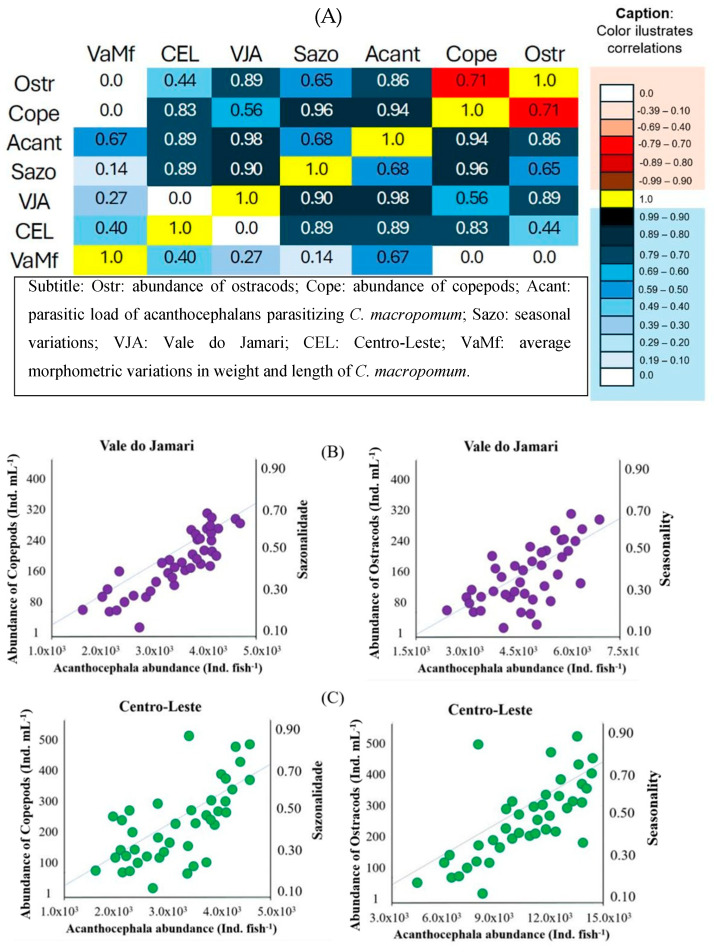
Correlations between the parasitic load of acanthocephalans, the morphometric variations in the host fish, the seasonality and abundances of ostracods and copepods (**A**), and specific correlations for the microregions of Vale do Jamari (**B**) and Centro-Leste of Rondônia state (**C**).

The abundances of ostracods and copepods showed a significant inverse correlation (r = 0.71; *p* = 0.018), indicating interspecific competition between these zooplankton groups within the water column of fishponds. Both ostracod (r = 0.86; *p* = 0.014) and copepod (r = 0.94; *p* = 0.012) abundances exhibited an intensive correlation with the acanthocephalan parasitic load, suggesting that acanthocephalans may use these zooplankton groups as hosts during some phase of their life cycles within the fishpond environment. Seasonal variations were significantly correlated with the abundances of ostracods (r = 0.65) and copepods (r = 0.96), as well as with the acanthocephalan parasitic load (r = 0.68). However, no significant correlation was found with the average weight and length of *C. macropomum* (r = 0.14; *p* > 0.05) (Figure 4A). When analyzing correlations by microregion, in the Vale do Jamari, the acanthocephalan parasitic load showed a higher intensive correlation with ostracods (r = 0.74) than with copepods (r = 0.44) (Figure 4B). In contrast, in the Centro-Leste microregion, the acanthocephalan parasitic load showed a higher intensive correlation with copepods (r = 0.57) than with ostracods (r = 0.45) (Figure 4C).

The stomachs and the anterior and posterior intestines of 196 *C. macropomum* belonging to the 41 properties visited were inspected. *N. buttnerae* were identified and quantified in 163 *C. macropomum*, with a higher incidence of infection in the anterior intestine with 27,446 parasites, followed by the posterior intestine with 15,446, and the stomach with 311. The prevalence rate of *N. buttnerae* infection in the region of study was 80.61%, with an average intensity of 273.3 Acanthocephala per *C. macropomum* and the abundance of 220 parasites (Table 4). In relation to hydrological seasons, the prevalence of infection was higher in the rainy season (91.67%), with the average intensity of Acanthocephala (236.85) being lower than that observed in the dry season (286.61). The category of adult fish showed the highest prevalence of infection (86.11%) compared to the other categories (Table 4) and the average intensity was higher in fish classified in the finishing category. *C. macropomum* in the juvenile category had the lowest prevalence and abundance rates compared to the other categories.

At the same time, *C. macropomum* in the juvenile category (100 to 500 g) showed the best condition factor Kn 1.01 without parasitic infestation (<1) and the condition reduced according to parasite intensity, 0.89 (1 to 300) and 0.69 (>300), respectively. However, despite the highest parasite intensity (>300) showing a greater total weight of 343.13 g and spleen weight of 0.45 g, there was no variation in the total length and weight of the liver (*p* > 0.05). Meanwhile, the HSI 2.34 and SSI 0.41 showed the highest averages under parasite intensity (1 to 300; *p* > 0.05). Adults (501 to 1500 g) showed the lowest condition factor of 0.82 for parasite intensity (>300). Furthermore, under parasite intensity (>300) the HSI 2.96 showed the highest average (*p* < 0.05), while total weight, total length, spleen weight, liver weight, and the SSI index did not vary under parasite intensity (*p* > 0.05). While *C. macropomum* ready for commercialization (>1501 g) presented the lowest condition factor of 0.86 under parasite intensity (>300), they showed no variation in total length between parasite intensities (*p* > 0.05). However, total weight 2.749 g, spleen weight 2.85 g, and liver weight 53.56 g showed higher means for parasite intensity (1 to 300; *p* < 0.05). Regarding the HSI 1.94 and RES 0.14 indices, they showed the highest averages without parasitic infestation (<1; *p* < 0.05) (Table 5).

The number of *C. macropomum* examined was 35 juveniles (100 to 500 g), 72 adults (501 to 1500 g), and 90 fish for commercialization (>1501 g). Juveniles weighed an average of 331 g, adults 987 g, and fish ready for commercialization 2497 g. Finishing *C. macropomum* had the highest average total length of 49.40 cm, prevalence of 78.80%, the highest parasite intensity index of 2373, the highest average intensity of 357, and the highest average abundance of 285.35 parasites per fish (Table 6).

**Figure 5:**
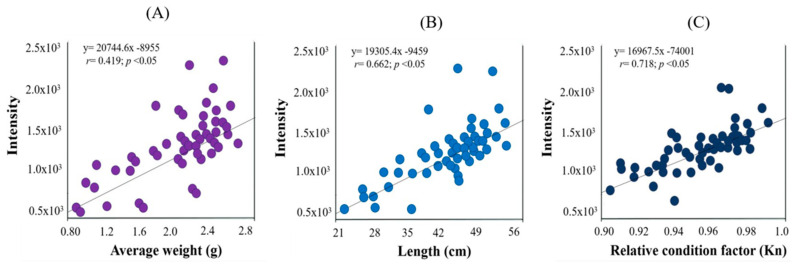
Correlations between weight (**A**), length (**B**), relative condition factor Kn (**C**), and parasite load (number of parasites/fish) of acanthocephalans parasitizing *Colossoma macropomum* in the Vale do Jamari and Centro-Leste microregions of Rondônia state.

The weight of *C. macropomum* showed a low correlation with parasite intensity (r = 0.419) (Figure 5A); however, the length of *C. macropomum* showed a significant correlation with parasite intensity (r = 0.662) (Figure 5B). Meanwhile the relative condition factor of the fish showed a strong correlation with parasite intensity (r = 0.718) (Figure 5C).

### 3.2. Physicochemical Parameters of Water and Sanitary Management

The values found for the water quality variables agreed with the parameters suitable for the cultivation of *C. macropomum* during the rainy and dry seasons in Vale do Jamari and in the Centro-Leste microregions of Rondônia state. In the Vale do Jamari, higher transparency (41.33 cm) and total ammonia (0.14 mg L-N-NH_4_) averages were found in the dry season (*p* < 0.05), while alkalinity (36.06 mg L^−1^ CaCO_3_) and hardness (30.85 mg L^−1^ CaCO_3_) averages were higher in the rainy season (*p* < 0.05). The other parameters did not show a significant difference between the seasons. In Centro-Leste, averages of dissolved oxygen (6.13 mg L^−1^), transparency (45.16 cm), nitrate (0.33 NO_3_^1−^), nitrite (0.13 NO_2_^1−^), and alkalinity were found (46.79 mg L^−1^ CaCO_3_) to be higher in the rainy season (*p* < 0.05). While the averages of phosphate (0.81 PO_4_^3−^) and phosphorus (0.80 P) were higher in the dry season (*p* < 0.05), the other parameters did not show a significant difference between the seasons (Table 7).

**Figure 6:**
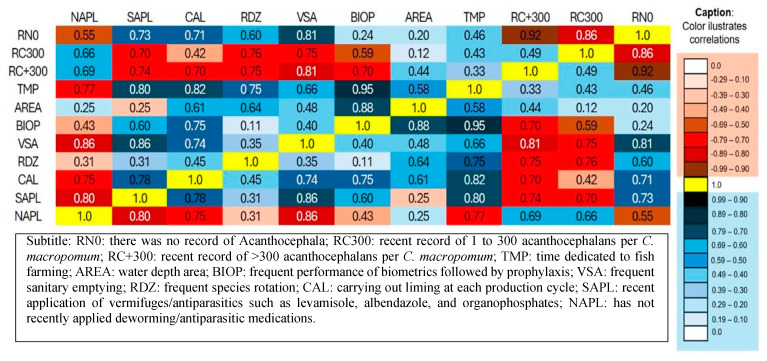
Correlations between health management practices and records of acanthocephalans parasitizing *Colossoma macropomum*.

The average water depth of the fish farms visited is 11.1 hectares. The non-occurrence of acanthocephalans parasitizing *C. macropomum* had a significant correlation with the applications of liming (r = 0.71), species rotation (r = 0.60), and sanitary emptying (r = 0.81). While records of acanthocephalans at different intensities showed a negative correlation with these sanitary management variables—liming (>300 r = −0.70), species rotation (>300 r = −0.75; 1–300 r = −0.76), and sanitary void (>300 r = −0.81; 1–300 r = −0.75). In relation to the recent applications of dewormers/antiparasitics, they showed a significant correlation with the lack of registration of parasitic infestations (r = 0.73), dedication time (r = 0.80), biometrics followed by prophylaxis (r = 0.60), sanitary emptiness (r = 0.86), and liming (r = 0.78). Meanwhile, the non-application of dewormers/antiparasitics showed a significant correlation with records of parasitic infestations (>300 r = 0.66 and 1–300 r = 0.69), as well as negative correlations with dedication time (r = −0.77), sanitary void (r = −0.86) and liming (r = −0.75) (Figure 6).

**Figure 7:**
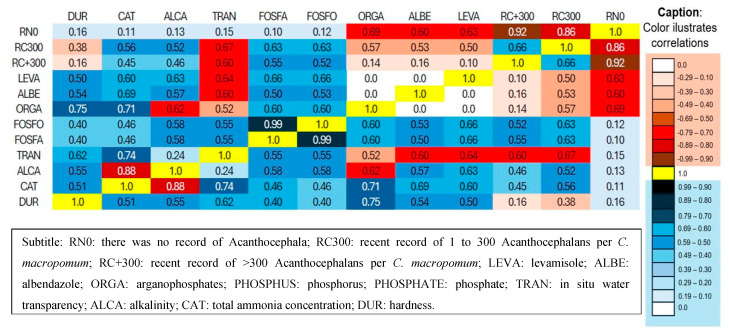
Correlations between dewormers/antiparasitics and the parasite intensity (no parasites, 1 to 300 and >300) of acanthocephalans in *Colossoma macropomum* and water quality variables.

The absence of RN0 parasites did not significantly influence the water quality variables (*p* > 0.05), while it showed a significant inverse correlation with deworming/antiparasitic drugs (organophosphates, r = 0.69; albendazole, r = 0.60; and levamisole, r = 0.63). Parasite intensity 1 to 3 Acanthocephalus RC300 did not significantly influence water quality variables (*p* > 0.05), except transparency (r = 0.67). Furthermore, RC300 did not show a significant correlation with deworming applications (*p* > 0.05). Finally, parasite intensity > 300 acanthocephalans RC + 300 did not significantly influence water quality variables (*p* > 0.05), except transparency (*p* < 0.60) (Figure 7). The application of dewormers influenced water quality variables. Levamisole showed a significant correlation with total ammonia concentration (r = 0.60) and alkalinity (r = 0.63) and an inverse correlation with transparency (r = −0.64). Regarding the application of albendazole, a significant correlation was found with the concentration of total ammonia (r = 0.69) and a significant inverse correlation with transparency (r = 0.60). Meanwhile, the application of organophosphate dewormers showed a significant correlation with hardness (r = 0.75), concentration of total ammonia (r = 0.71), phosphorus (r = 0.60), and phosphate (0.60), as well as a significant inverse correlation with alkalinity (r = 0.62) (Figure 7)

## 4. Discussion

Aquaculture plays a crucial role in meeting global food demands while promoting economic development. However, ensuring the proper growth and survival of aquatic organisms requires effective environmental management, particularly regarding water quality. Key variables such as temperature, pH, dissolved oxygen, and nutrient levels must be consistently monitored to prevent adverse conditions that could impair fish health, reduce productivity, or even lead to mass mortality. Poor water management can create favorable conditions for parasites and pathogens, which thrive under stressful environments caused by overcrowding, poor nutrition, or degraded water quality.

One prominent challenge in aquaculture is the proliferation of parasites, such as *Neoechinorhynchus buttnerae*, which affect farmed species like *Colossoma macropomum*. These parasites exploit intermediate hosts like ostracods, perpetuating infestations in fishponds, particularly under high organic matter accumulation. Such conditions can severely impact fish growth, compromise production yields, and jeopardize the economic feasibility of aquaculture activities.

This study highlights the parasite–environment relationship, examining water quality, plankton dynamics, and parasitic infestations in aquaculture systems. By addressing gaps in parasitic ecology and evaluating management strategies, the research aims to contribute to the socioeconomic and environmental sustainability of aquaculture. The findings provide valuable insights to optimize farming conditions, enhance sanitary practices, and ensure the long-term viability of fish farming, particularly in regions like Rondônia, Brazil.

In the pursuit of the proper development of aquatic organisms and the economic feasibility of farming, while aiming for the socioeconomic and environmental sustainability of this productive activity, it is essential to control the environment used for this purpose, i.e., the water utilized in aquaculture. Some of the main water quality variables to be periodically monitored include temperature, color, turbidity, transparency, pH, alkalinity, hardness, dissolved oxygen, phosphorus, ammonia, nitrite, and nitrate. When these variables fall outside the appropriate parameters for the cultivated species, they can reduce reproductive and productive performance as well as survival, potentially jeopardizing the viability of the activity and, in extreme cases, leading to the mass mortality of the farmed animals [16].

The lack of proper management practices, in addition to causing various problems, can also contribute to the establishment of parasites and pathogens, which can become a serious issue, compromising development, survival, and consequently economic outcomes, endangering the viability of the activity. A significant management problem, capable of jeopardizing the entire production, is the lack of disinfection procedures between farming cycles [17]. Currently, information is available on performing analyses and corrections that can provide a suitable environment, optimizing the development of farmed animals and directly and positively impacting the outcomes of this activity. Regarding issues related to parasite and pathogen control, although some literature exists, many gaps remain that need to be urgently filled given the potential catastrophic consequences of this lack of information, as it impacts not only economic but also environmental and social aspects.

Economic costs related to disease-induced mortality in Brazil, estimated through a model using official data on the production and mortality of bony fish and considering both direct and indirect economic costs for freshwater fish farming, amounted to USD 84 million per year [18]. The adoption of good sanitary management practices in production, as well as avoiding the introduction of parasites and their intermediate hosts into the cultivation fishponds, is the best way to control diseases. Furthermore, it is essential to prevent the introduction of potentially parasite-contaminated water into the fishponds when acquiring new fish; disinfect the production system (ponds and handling equipment); maintain good water quality in the cultivation ponds, with satisfactory levels of dissolved oxygen; and ensure proper nutrition and feeding management [19].

According to Cunha et al. [20], in the aquatic environment, fish are subject to various environmental changes that cause stress, potentially increasing their vulnerability to pathogens. The most studied taxa include the groups Protozoa, Myxozoa, Monogenea, Digenea, Cestode, Nematoda, Acanthocephala, and Crustacea, which are excellent biological indicators of environmental quality. Their populations tend to increase or decrease in response to changes in water quality, particularly ectoparasites, which are considered excellent indicators due to their monoxenic life cycle, high reproductive rates, and immediate response to changes in the aquatic biota. Parasites with a heteroxenic life cycle show population alterations related to the death of their intermediate hosts, while endoparasites such as Digenea, Acanthocephala, Nematoda, and Cestoda are frequently studied in relation to the environment [21].

Jerônimo et al. [5] state that the spread of these organisms is often favored when animals are under stressful conditions, such as high stocking densities, abrupt temperature fluctuations, inadequate feeding, and high concentrations of organic matter in the water. They explain that these factors, when uncontrolled, cause homeostatic imbalances in fish, increasing their susceptibility to pathogens. Castro et al. [22] mention that various groups of parasites have been identified in Brazilian *C. macropomum*, including protozoa, myxosporeans, crustaceans, and helminths, which can proliferate and cause diseases when conditions are favorable.

The lack of sanitary maintenance in cultivation ponds, particularly concerning water quality, can promote the existence of an environment favorable to the life cycle of undesirable organisms. The absence of good management practices, such as using appropriate stocking densities and implementing sanitary practices between cycles, such as draining and disinfecting fishponds, can favor the development of acanthocephalans in the system. In this sense, eliminating the intermediate host from the environment can be a strategy for controlling this endoparasite [17].

The accumulation of organic matter, whether through leaching and surface runoff or the excessive input of nutrients via fertilization and feeding, can degrade the ideal environmental conditions for farmed fish, favoring the development of undesirable organisms. Parasites such as acanthocephalans may benefit from this condition, ensuring their perpetuation through eggs deposited in organic matter or by infecting other aquatic organisms (ostracods or copepods) that host the young form of the parasite (acanther). According to Malta et al. [7], “due to the zooplanktophagous feeding habit of *C. macropomum* and because the intermediate hosts of *N. Buttnerae* (Acanthocephala) are part of the plankton, the life cycle of this acanthocephalan species was completed in the farms, causing these massive infestations”, or by parasitizing the residual farmed fish from the previous crop, causing losses to the aquaculture activity.

It was observed that *N. buttnerae* undergoes development after the egg is predated by an ostracod, with the stages being acanthor, acanthella (with eight developmental changes), and cystacanth, the last of which is the infectious stage, reached after 29 days. Ostracods reproduce sexually, generating resistant eggs that can survive for long periods (up to decades) in dry soil environments, hatching when rehydrated and exposed to light. It is known that liming techniques using lime, CaO, or hydrated lime Ca(OH)_2_, widely used by fish farmers in Brazil, seem ineffective in eliminating the ostracod *C. vidua*, identified as the intermediate host of *N. buttnerae*. This highlights the importance of understanding the intermediate host of this parasite and its life cycle as a useful tool for preventing and combating infections caused by this parasite [23].

Chagas et al. [24] point out that the intensification of production systems is directly related to the occurrence of parasitic diseases, especially acanthocephalans, particularly due to the high infestations recorded in the northern region of Brazil. They state, “High infestations of acanthocephalans are responsible for the partial and total occlusion of the intestinal tract, impairing absorption capacity and directly competing with ingested food. Fish growth is compromised by the parasitosis, and they become more susceptible to common aquaculture management practices.”

The phylum Acanthocephala consists of helminths exclusively parasitic in the intestines of vertebrates, with nine species occurring in Amazonian fish, two of which are capable of parasitizing *C. macropomum—Echinorhynchus jucundus* of the family Echinorhynchidae and *Neoechinorhynchus buttnerae* Golvan, 1956, of the Neoechinorhynchidae. The same author further states that ostracods, copepods, and the larvae of Megaloptera are the intermediate hosts of acanthocephalans from the Neoechinorhynchidae family [7]. In this study, the mean intensity of *N. buttnerae* was consistent with studies on farmed *C. macropomum* in the states of Tocantins, 19 to 234 [17]; Roraima, 188 to 388 [25]; and Amazonas, 31 to 406 [7]; 81 to 708 [26]; 15 to 720 [4]; 107 to 921 [23]; and 54 to 931 and 81 to 1,219 [24].

The research developed considered the “socioeconomic–environmental” aspect, aiming to generate information and knowledge that could assist in improving farming conditions through biological and physicochemical evaluations of the cultivation water, contributing to the pursuit of sustainability in its various aspects, and thus supporting the viability of this important activity. Studies conducted to investigate the potential of the parasite as an environmental quality bioindicator can be a useful tool, utilizing information on parasitic ecology and the consequent maintenance of sanitary aspects in confined environments. In this context, the literature on aquaculture is scarce when compared to that of the natural environment [5,27].

It is worth noting that the availability of this information is of great value in the pursuit of food and environmental security, as well as the sustainability of the activity. The study provides information on the parasite–environment relationship, presenting a survey of the parasitic fauna of *C. macropomum* and its relationship with water quality in aquaculture farms in Rondônia, focusing on the biological aspect. This was achieved by evaluating plankton and its relationship to parasitism, as well as the control strategies used.

## 5. Conclusions

It is possible to use the presence of zooplankton ostracods and copepods to predict possible parasitic infestations in *Colossoma macropomum* farmed. These zooplankton are like sentinels, strategic and sensitive bioindicators capable of assessing the health of the aquatic ecosystem. Although there are few studies exploring this approach in fish farms compared to natural environments, acanthocephalans have been mainly linked to water quality parameters in fish farms rather than specific pollutants.

Research around ecotoxicology is gaining prominence, demanding collaboration between parasitologists and toxicologists. Endoparasites found naturally in the aquatic environment have been shown to be valuable indicators of toxic accumulation, especially of heavy metals. In fish farms, zooplankton and parasites can be indicators of adverse effects, especially when changes in parasite communities are monitored. This approach is particularly advantageous for detecting changes in artificial ecosystems that could have significant long-term impacts.

## Figures and Tables

**Table 1:** Municipality of collections, average length (SD±) in cm, average weight (g), and number of fish per property in the two Amazon hydrographic seasons.

			Rainy		Dry
Properties/Municipalities		Weight (g)	SD± (cm)	Weight (g)	SD± (cm)
Number of fish examined					
P1Ji-Paraná	6	668	33.0	673	30.0
P2Ji-Paraná	6	2315	47.2	2596	49.8
P3Ji-Paraná	6	2031	47.6	851	37.1
P4 Presidente Medici	6	978	37.3	2399	51.3
P5Urupá	6	2860	52.3	661	32.6
P6Urupá	5	1145	37.0	560	31.5
P7Urupá	4	1885	43.6	1390 *	45 *
P8Urupá	6	298	24.6	955	37.8
P9Urupá	3	893	35.6	-	-
P10Urupá	3	691	35.0	-	-
P11Vale do Paraíso	6	3431	62.5	173.3	21.3
P12Vale do Paraíso	3	3033	52.0	-	-
P13Ouro Preto D’ Oeste	6	1105	39.0	1475	43.0
P14Texeiropolis	6	2390	51.0	3336	52.3
P15Ouro Preto D’ Oeste	6	756	37.1	1007	37.3
P16Vale do Paraíso	6	1937	48.0	1280	40.6
P17Nova União	6	3103	54.6	1916	49.1
P18Nova União	5	1556	38.6	987.5	36.0
P19Mirante da Serra	4	1580	41.5	1560 *	46.0 *
P20Mirante da serra	3	1035	38.5	-	-
P21Urupá	3	-	-	2021	46.0
P22Ouro Preto D’ Oeste	3	-	-	2180	38.3
P24Ariquemes	6	2053	41.6	1073	38.6
P25Machadinho D’ Oeste	3	-	-	765	38.0
P26Machadinho D’ Oeste	3	-	-	2573	51.6
P27Cacaulândia	6	1755	41.5	816.6	40.0
P28Cacaulândia	3	-	-	1408	43.5
P29Monte Negro	6	871.6	34.6	2298	51.0
P30Buritis	6	1249	41.0	291	26.6
P31Buritis	6	3426	28.4	2011	48.3
P32Buritis	6	1078	38.5	1570	44.6
P33Ariquemes	6	1423	44.0	908.3	42.0
P34Texeiropolis	3	-	-	1543	45.6
P35Presidente Medici	1	-	-	1465	44.0
P36Vale do Paraíso	3	1577	42.0	-	-
P37Vale do Paraíso	3	-	-	1802	45.0
P38Ariquemes	3	-	-	2341	45.0
P39Ariquemes	6	1805	43.3	741.6	33.5
P40Ariquemes	6	2260	45.9	1908	40.0
P41Rio Crespo	6	1283	44.8	2160	47.8
P42Rio Crespo	6	3626	55.3	1288	36.9

* Only one fish examined.

**Table 2:** Properties visited by municipalities in Rondônia state, total number, and mean and standard deviation (SD±) of *Neoechinorhynchus buttnerae* (Acanthocephala) quantified in *Colossoma macropomum* in the two hydrological seasons (rainy and dry).

	Rainy		Dry	
Properties/Municipalities	No. of Acanthocephala found	Average/SD±	No. of Acanthocephala found	Average/SD±
P1 Ji-Paraná	538	179.33 (±145.38)	0	0
P2 Ji-Paraná	1203	401.00 (±268.24)	1924	641.33 (±268.24)
P3 Ji-Paraná	1649	549.66 (±295.57)	137	68.50 (±64.35)
P4 Presidente Médici	2624	549.66 (±295.57)	450	150.00 (±92.82)
P5 Urupá	682	227.33 (±204.58)	803	267.66 (±192.08)
P6 Urupá	75	37.5 (±24.74)	83	41.50 (±31.81)
P7 Urupá	*238	--	*223	--
P8 Urupá	0	0	399	133.00 (±122.50)
P9 Urupá	133	44.33 (±41.86)	*--	--
P10 Urupá	203	67.66 (±39.71)	*--	--
P11 Vale do Paraíso	1782	594.00 (±749.53)	285	95.00 (±177.48)
P12 Vale do Paraíso	1887	629.00 (±298.08)	--	--
P13 Ouro Preto do Oeste	289	144.50 (±96.87)	92	--
P14 Texeirópolis	1659	553.00 (±546.50)	1366	455.33 (±287.81)
P15 Ouro Preto do Oeste	435	145.00 (±179.18)	532	177.33 (±216.92)
P16 Vale do Paraíso	639	213.00 (±324.03)	70	23.33 (±30.17)
P17 Nova União	1214	404.66 (±243.82)	1252	417.33 (±420.71)
P18 Nova União	37	12.33 (±7.57)	1025	512.00 (±611.64)
P19 Mirante da Serra	349	--	349	--
P20 Mirante da Serra	46	23.00 (±16.97)	--	--
P21 Urupá	--	--	*0	0
P22 Ouro Preto do Oeste	--	--	31	15.50 (±16.26)
P23 Ariquemes	488	244.00 (±125.86)	*0	0
P24 Machadinho do Oeste	--	--	236	402.66 (±42.00)
P25 Machadinho do Oeste	*0	0	1208	402.66 (±449.32)
P26 Cacaulândia	462	152.00 (±157.12)	1622	540.66 (±508.09)
P27 Cacaulândia	--	--	114	--
P28 Monte Negro	1084	542.00 (±504.87)	18	--
P29 Buritis	99	33.00 (±5.57)	1166	388.66 (±101.54)
P30 Buritis	15	5.00 (±4.00)	14	7.00 (±0.00)
P31 Buritis	2977	992.33 (±126.88)	958	319.33 (±342.06)
P32 Ariquemes	218	72.66 (±101.10)	2010	670.00 (±625.09)
P33 Texeirópolis	--	--	58	29.00 (±33.94)
P34 Presidente Médici	--	--	*0	0
P35 Vale do Paraíso	2081	693.00 (±195.79)	--	--
P36 Vale do Paraíso	--	--	254	84.66 (±10.32)
P37 Ariquemes	--	--	254	84.66 (±10.43)
P38 Ariquemes	*0	0	55	27.00 (±2.47)
P39 Ariquemes	33	11.00 (±0.87)	355	118.33 (±16.77)
P40 Rio Crespo	33	11.00 (±0.87)	355	118.33 (±16.77)
P41 Rio Crespo	1413	471.00 (±468.60)	1300	433.33 (±35.49)
Total	14,474	185.54 (±167.52)	11,671	124.98 (±176.45)

**Table 3:** Abundances of copepod and ostracod species (Ind. L^−1^) under conditions of parasitic intensities—non-intense (<300 acanthocephalans per *Colossoma macropomum*) and intense (>300 acanthocephalans per *C. macropomum*)—in the Vale do Jamari and Centro-Leste microregions of Rondônia state, Brazil.

Parasite Intensity					Microregions of Rondônia		
		<300	>300	*p* Value	Vale do Jamari	Centro- Leste	*p* Value
Species	Order						
*Thermocyclops* *decipiens* (Kiefer, 1929)	Cyclopoida	133.66 (±11.36) **^b^**	444.20 (±32.43) **^a^**	0.011 *	144.21 (±8.65) **^b^**	496.50 (±27.31) **^a^**	0.003 *
*Acanthocyclops* sp. (Kiefer, 1929)	Cyclopoida	0.10 (±0.01) **^b^**	226.82 (±16.56) **^a^**	0.005 *	16.20 (±0.97) **^b^**	344.75 (±18.96) **^a^**	0.006 *
*Argyrodiaptomus* sp. (Brehm, 1933)	Calanoida	10.99 (±0.93) **^b^**	198.00 (±14.45) **^a^**	0.009 *	38.65 (±2.32) **^b^**	108.22 (±5.95) **^a^**	0.009 *
*Argyrodiaptomus furcatus* (Sars G.O. 1901)	Calanoida	0.64 (±0.05) **^b^**	16.22 (±1.18) **^a^**	0.018 *	4.30 (±0.26) **^b^**	96.80 (±5.32) **^a^**	0.011*
**Copepods**	**-**	**155.39** (±13.21) **^b^**	**885.24** (±64.62) **^a^**	0.015 *	**203.36** (±12.20) **^b^**	**1046.27** (±57.54) **^a^**	0.013 *
*Heterocypris* sp. (Claus, 1892)	Podocopida	122.08 (±10.37) **^b^**	664.10 (±48.47) **^a^**	0.019 *	898.64 (±53.90) **^a^**	80.20 (±4.41) **^b^**	0.003 *
*Heterocypris punctata* (Keyser, 1975)	Podocopida	25.25 (±21.15) **^b^**	100.21 (±7.31) **^a^**	0.037 *	303.16 (±18.19) **^a^**	20.00 (±1.10) **^b^**	0.008 *
**Ostracods**	**-**	**147.33** (±12.52) **^b^**	**764.31** (±55.79) **^a^**	0.012 *	**1201.80** (±61.31) **^a^**	**100.20** (±5.51) **^b^**	0.002 *

* If there are different letters (a, b), the means of parasitic infestations and microregions are different according to Student’s *t*-test (*p* < 0.05).

**Table 4:** Number of *Colossoma macropomum* examined, prevalence, average intensity, and abundance in the study areas by hydrological season and fish weight categories.

	*C. macropomum* Examined	Prevalence (%)	Average Intensity	Abundance
In 41 fish farms	196	80.61	273.36	220
Rainy season	96	91.60	236.85	217.11
Dry season	100	75.00	286.61	214.96
Juveniles (100 to 500 g)	35	69.00	210.00	146.00
Adultes (501 a 1500 g)	72	86.11	206.80	178.11
Ready for commercialization (>1501 g)	90	78.80	357.00	285.35

**Table 5:** Morphometric variations and organ weights, hepatosomatic (HSI) and splenosastic (SSI), and the condition factor (Kn) according to parasite intensity in the juvenile (100 to 500 g), adult (501 to 1500 g), and ready for commercialization (>1501 g) categories.

^#^ Parasite Intensity	Juveniles (100 to 500 g)				Adults (501 to 1500 g)				Ready for Commercialization (>1501 g)			
	<1	1 to 300	>300	*p* Value	<1	1 to 300	>300	*p* Value	<1	1 to 300	>300	*p* Value
Total weight (g)	305.00 (±35.53) **^b^**	358.00 (±46.58) **^a^**	343.13 (±17.16) **^a^**	0.03 *	977.00 (±113.82) ^a^	953.00 (±123.89) ^a^	894.00 (±44.70) ^a^	> 0.05	2102.00 (±244.88) **^b^**	2749.00 (±357.37) **^a^**	2387.00 (±119.35) **^b^**	0.03 *
Total length (cm)	26.39 (±1.22) ^a^	28.03 (±0.99) ^a^	27.50 (±1.47) ^a^	> 0.05	38.56 (±1.81) ^a^	37.50 (±1.33) ^a^	38.63 (±2.07) ^a^	> 0.05	48.30 (±2.26) ^a^	52.00 (±1.84) ^a^	50.10 (±2.68) ^a^	> 0.05
Spleen (g)	0.28 (±0.01) **^b^**	0.26 (±0.01) **^b^**	0.45 (±0.01) **^a^**	0.04 *	0.94 (±0.04) ^a^	0.99 (±0.70) ^a^	1.14 (±0.07) ^a^	> 0.05	2.23 (±0.04) **^c^**	2.85 (±0.05) **^a^**	2.59 (±0.03) **^b^**	0.01 *
Liver (g)	4.50 (±0.20) ^a^	2.34 (±0.16) ^a^	4.84 (±0.29) ^a^	> 0.05	20.67 (±1.14) **^b^**	20.11 (±1.21) **^b^**	25.54 (±1.53) **^a^**	0.02 *	45.11 (±2.00) **^b^**	53.56 (±3.78) **^a^**	45.8 (±2.75) **^b^**	0.02 *
HSI ^1^	1.36 (±0.21) **^b^**	2.34 (±0.31) **^a^**	1.40 (±0.20) **^b^**	0.04 *	1.59 (±0.25) **^c^**	2.08 (±0.27) **^b^**	2.96 (±0.43) **^a^**	0.04 *	3.54 (±0.57) **^a^**	1.94 (±0.26) **^b^**	1.84 (±0.27) **^b^**	0.04 *
SSI ^2^	0.11 (±0.01) **^b^**	0.41 (±0.04) **^a^**	0.14 (±0.02) **^b^**	0.04 *	0.10 (±0.01) ^a^	0.10 (±0.01) ^a^	0.13 (±0.02) ^a^	> 0.05	0.14 (±0.02) **^a^**	0.09 (±0.01) **^b^**	0.10 (±0.01) **^b^**	0.04 *
Kn ^3^	1.01 (±0.05) ^a^	0.8 (±0.05) ^b^	0.69 (±0.03) ^c^	0.04 *	0.97 (±0.05) **^b^**	1.09 (±0.05) **^a^**	0.82 (±0.04) **^c^**	0.04 *	0.95 (±0.05) **^b^**	1.11 (±0.05) **^a^**	0.86 (±0.04) **^c^**	0.01 *

* If there are different letters (a, b, c), the means are different according to Tukey’s test (*p* < 0.05); ^#^ Intensities of Acantocephala infestation by *C. macropomum*; ^1^ HSI: hepatosomatic relationship index; ^2^ SSI: splenosomatic relationship index; ^3^ Kn: condition factor of parasitized *C. macropomum*.

**Table 6:** Prevalence (%), intensity, average intensity, and parasite abundance in *Colossoma macropomum* juveniles (100 to 500 g), adults (501 to 1500 g), and fish ready for commercialization (>1501 g).

		Weight Classes		
Variáveis	Juveniles (100 a 500 g)	Adults (501 a 1500 g)	Ready for commercialization (>1501 g)	*p* value
*C. macropomum* examined	35	72	90	
Total weight (g)	331.00 (±18.21) **^c^**	987.00 (±54.29) **^b^**	2497.00 (±137.34) **^a^**	0.014 *
Total length (cm)	27.01 (±2.97) **^c^**	38.50 (±4.24) **^b^**	49.40 (±5.43) **^a^**	0.016 *
Prevalence (%)	69.00 (±6.21) **^b^**	86.11 (±7.76) **^a^**	78.80 (±7.09) **^a^**	0.031 *
Intensity	919.00 (±128.66) **^b^**	2054.00 (±287.56) **^a^**	2373.00 (±332.22) **^a^**	0.011 *
Average intensity	210.00 (±26.04) **^b^**	206.80 (±25.64) **^b^**	357.00 (±44.27) **^a^**	0.025 *
Abundance	146.00 (±11.68) **^b^**	178.11 (±14.25) **^b^**	285.35 (±22.83) **^a^**	0.033 *

* If there are different letters (a, b, c) the means are different according to Tukey’s test (*p* < 0.05).

**Table 7:** Average and SD± of the physicochemical variables of water from fish farms located in the Vale do Jamari and Centro-Leste microregions in the rainy and dry seasons.

			Microregions			
Variables	Vale do Jamari		*p* value	Centro-Leste		*p* value
	rainy	dry		rainy	dry	
Temperature (°C)	29.42 (±1.62) ^a^	29.99 (±1.65) ^a^	>0.05	27.66 (±1.66) ^a^	30.10 (±1.81) ^a^	>0.05
pH	7.50 (±0.50) ^a^	7.27 (±0.49) ^a^	>0.05	6.41 (±0.46) ^a^	7.37 ± (0.52) ^a^	>0.05
Dissolved oxygen (mg L^−1^)	6.05 (±0.12) ^a^	5.79 (±0.11) ^a^	>0.05	**6.13 (±0.12) ^a^**	**3.13 (±0.06) ^b^**	**0.010**
Transparency (cm)	**41.33 (±0.61) ^b^**	**55.23 (±0.82) ^a^**	**0.017**	**45.16 (±0.77) ^a^**	**35.44 (±0.60) ^b^**	**0.016**
1- Nitrate (NO_3_)	1.40 (±0.28) ^a^	0.96 (±0.19) ^a^	>0.05	**0.33 (±0.07) ^a^**	**0.15 (±0.03) ^b^**	**0.330**
1- Nitrite (NO_2_)	0.07 (±0.01) ^a^	0.05 (±0.01) ^a^	>0.05	**0.13 (±0.03) ^a^**	**0.05 (±0.01) ^b^**	**0.037**
Total ammonia (mg L^−^N-NH_4_)	**0.06 (±0.01) ^b^**	**0.14 (±0.03) ^a^**	**0.038**	0.10 (±0.02) ^a^	0.13 (±0.03) ^a^	>0.05
Phosphate (PO_4_^3−^)	0.75 (±0.02) ^a^	0.79 (±0.20) ^a^	>0.05	**0.81 (±0.24) ^b^**	**1.96 (±0.59) ^a^**	**0.025**
Phosphorus (P)	0.24 (±0.05) ^a^	0.26 (±0.05) ^a^	>0.05	**0.32 (±0.08) ^b^**	**0.80 (±0.20) ^a^**	**0.029**
Alkalinity (mg L^−1^ CaCO_3_)	**36.06 (±2.89) ^a^**	**30.29 (±2.42) ^b^**	**0.024**	**46.79 (±3.27) ^a^**	**35.51 (±2.48) ^b^**	**0.015**
Hardness (mg L^−1^ CaCO_3_)	**30.85 (±2.37) ^a^**	**24.79 (±1.91) ^b^**	**0.020**	52.67 (±4.63) ^a^	32.60 (±2.87) ^b^	>0.05

If there are different letters (a, b) between the rainy and dry seasons, the means are different according to Student’s *t*-test (*p* < 0.05).

## Data Availability

Data are contained within the article.

## References

[1] FAO, IFAD, UNICEF, WFP, WHO (2022). The State of Food Security and Nutrition in the World 2022.

[2] Rocha A.S.C.M., Baldi S.C.V., de Souza M.L.R., Rosa B.L., Soares E.C., Pontuschka R.B., Dantas Filho J.V., Cavali J. (2023). Proximate composition, energy value, and lipid quality in loin in different weight classes of pirarucu (*Arapaima gigas*) from fish farming. Bol. Inst. Pesca.

[3] Peixe BR (2023). Anuário 2023 da Peixe BR da Piscicultura.

[4] Silva-Gomes A.L., Coelho Filho J., Viana-Silva W., Braga-Oliveira M.I., Bernardino G., Costa J.I. (2017). The impact of *Neoechinorhynchus buttnerae* (Golvan, 1956) (Eoacanthocephala: Neochinorhynchidae) outbreaks on productive and economic performance of the tambaqui *Colossoma macropomum* (Cuvier, 1818), reared in ponds. Lat. Am. J. Aquat. Res..

[5] Jerônimo G.T., da Cruz M.G., de Aguiar Bertaglia E., Furtado W.E., Martins M.L. (2022). Fish parasites can reflect environmental quality in fish farms. Rev. Aquac..

[6] Lucena C.F.P., Dantas Filho J.V., Ferreira A.M.S., Rocha A.S.C.M., Schons S.D.V., Gasparotto P.H.G. (2023). Outbreaks of *Neoechinorhynchus buttnerae* (Acanthocephala) infection in raised semi-intensively *Colossoma macropomum* in Theobroma, Rondônia state, Western Amazon. Acta Vet. Bras..

[7] Malta J.C.O., Gomes A.L.S., Andrade S.M.S., Varella A.M.B. (2001). Infestações maciças por acantocéfalos, *Neoechinorhynchus buttnerae* Golvan, 1956, (Eoacanthocephala: Neoechinorhynchidae) em tambaquis jovens, *Colossoma macropomum* (Cuvier, 1818) cultivados na Amazônia Central. Acta Amaz..

[8] Alvares C.A., Stape J.L., Sentelhas P.C., Gonçalves J.L.M., Sparovek G. (2013). Koppen’s climate classification map for Brazil. Meteorol. Zeitschrisft.

[9] INPE. Instituto Nacional de Pesquisas Espaciais Centro de Previsão de Tempo e Estudos Climáticos (CPTEC). Estação meteorológica de Ouro Preto do Oeste—RO: INPE/CPTEC, 2022.

[10] Costa R.L., Figueiredo F.M., Bay M., Queiroz C.B., Bay-Hurtado F. (2016). Qualitative analysis of phytoplankton in a Fish farming of Alvorada d’Oeste, Rondônia, Brazil. Acta Agronómica.

[11] de Lima Pinheiro M.M., Santos B.L.T., Dantas Filho J.V., Pedroti V.P., Cavali J., Dos Santos R.B., Nishiyama A.C.O.C., Guedes E.A.C., de Vargas Schons S. (2023). First monitoring of cyanobacteria and cyanotoxins in freshwater from fish farms in Rondônia state, Brazil. Heliyon.

[12] dos Santos M., Frisso R.M., e Silva V.D.S., e Silva L.D.S., Garcez J.R., dos Santos G.F.D., de Brito J.M., de Barros R. (2024). Parasitic fauna in tambaqui (*Colossoma macropomum*) from fish farms in the municipality of Rorainópolis-Roraima-Brazil. Rev. Obs. Econ. Latinoam..

[13] Hernández J.F.P. (2021). Zooplancton—Biodiversidad acuática del Sitio Demostrativo de Ecohidrología PHI-UNESCO DRMI-Sitio Ramsar Complejo Cenagoso de Zapatosa.

[14] Virgilio L.R., Lima F.D.S., Negreiros L., Takemoto R.M., Camargo L.M.A., Meneguetti D.U.O. (2021). Occurrence of *Neoechinorhynchus curemai* (Acanthocephala: Neoechinorhynchidae) in *Prochilodus nigricans* (Characiformes: Prochilodontidae), in southwestern Amazon. Acta Sci. Biol. Sci..

[15] de Sousa Lourenço F., Morey G.A.M., Pereira J.N., de Oliveira Malta J.C. (2017). Ocorrência de *Neoechinorhynchus* (*neoechinorhynchus*) *buttnerae* golvan, 1956 (acantocephala: Neochinorhynchidae) em *colossoma macropomum* (cuvier, 1818) (Characiformes: Serrasalmidae) provenientes de uma piscicultura da amazônia brasileira. Folia Amaz..

[16] dos Santos D.K.M., Kojima J.T., Santana T.M., de Castro D.P., Serra P.T., Dantas N.S.M., da Fonseca F.A.L., Mariúba L.A.M., Gonçalves L.U. (2020). Farming tambaqui (*Colossoma macropomum*) in static clear water versus a biofloc system with or without *Bacillus subtilis* supplementation. Aquac. Int..

[17] Maciel-Honda P.O., Sousa E.M.D., Costa-Fernandes T.D.O., Jesus F.H.R.D., Chagas E.C., Tavares-Dias M. (2023). First record of *Neoechinorhynchus buttnerae* and *Piscinoodinium pillulare* infection in *Colossoma macropomum* in the state of Tocantins, Brazil. Brazilian J. Vet. Parasitol..

[18] Tavares-Dias M., Martins M.L. (2017). An overall estimation of losses caused by diseases in the Brazilian fish farms. J. Parasit. Dis..

[19] Jerônimo G.T., Tavares-Dias M., Mariano W.S. (2015). Parasitos de peixes Characiformes e seus híbridos cultivados no Brasil. Aquicultura No Brasil: Novas Perspectivas.

[20] Cunha K.D.N., Domingues M.V., Cunha L.D.d.S., Nunes Z.M.P. (2021). Parasitic monogenoideans of *Sciades herzbergii* as bioindicators of environmental quality in amazonian estuarines ecosystems. Braz. J. Vet. Parasitol..

[21] Virgilio L.R., de Melo H.P.S., da Silva Lima F., Takemoto R.M., Camargo L.M.A., de Oliveira Meneguetti D.U. (2023). Fish endoparasite metacommunity in environments with different degrees of conservation in the western Brazilian Amazon. Parasitol. Res..

[22] Castro L.D.A., Jerônimo G.T., Silva R.M.D., Santos M.J., Ramos C.A., Porto S.M.D.A. (2020). Occurrence, pathogenicity, and control of acanthocephalosis caused by *Neoechinorhynchus buttnerae*: A review. Braz. J. Vet. Parasitol..

[23] Lourenço F.D.S., Morey G.A.M., Malta J.C.D.O. (2018). The development of *Neoechinorhynchus buttnerae* (Eoacanthocephala: Neoechinorhynchidae) in its intermediate host *Cypridopsis vidua* in Brazil. Acta Parasitol..

[24] Chagas E.C., Aquino-Pereira S.L., Benavides M.V., Brandão F.R., Monteiro P.C., Maciel P.O. (2019). *Neoechinorhynchus buttnerae* parasitic infection in tambaqui (*Colossoma macropomum*) on fish farms in the state of Amazonas. Bol. Inst. Pesca.

[25] Pereira J.N., Morey G.A.M. (2018). First record of *Neoechinorhynchus buttnerae* (Eoacantocephala, Neochinorhynchidae) on *Colossoma macropomum* (Characidae) in a fish farm in Roraima, Brazil. Acta Amaz..

[26] Matos L.V., de Oliveira M.I.B., Gomes A.L.S., da Silva G.S. (2017). Morphological and histochemical changes associated with massive infection by *Neoechinorhynchus buttnerae* (Acanthocephala: Neoechinorhynchidae) in the farmed freshwater fish *Colossoma macropomum* Cuvier, 1818 from the Amazon State, Brazil. Parasitol. Res..

[27] Finizola e Silva M., Van Passel S. (2020). Climate-Smart Agriculture in the Northeast of Brazil: An Integrated Assessment of the Aquaponics Technology. Sustainability.

